# Estimating the agreement between the metabolic rate calculated from prediction equations and from a portable indirect calorimetry device: an effort to develop a new equation for predicting resting metabolic rate

**DOI:** 10.1186/s12986-018-0278-7

**Published:** 2018-06-15

**Authors:** Eleni Pavlidou, Dimitris Petridis, Maria Tolia, Nikolaos Tsoukalas, Antigoni Poultsidi, Aristeidis Fasoulas, George Kyrgias, Constantinos Giaginis

**Affiliations:** 1 Department of Food Science and Nutrition, University of Aegean, Mitropoliti Ioakim 2, Myrina, Lemnos, 81440 Athens, Greece; 2 Department of Food Technology, Technological Educational Institute, 57400 Thessaloniki, Greece; 3 Department of Radiotherapy, School of Health Sciences, Faculty of Medicine, University of Thessaly, 41110 Biopolis, Larissa, Greece; 4 Department of Oncology, Veterans Hospital (NIMTS), 10 Monis Petraki, 11521 Athens, Greece; 5 Surgery Clinic, School of Health Sciences, Faculty of Medicine, University of Thessaly, 41110 Larissa, Greece

**Keywords:** Basal metabolic rate, Indirect calorimetry, Predictive equation, Resting energy expenditure, Resting metabolic rate

## Abstract

**Background:**

Many studies have been performed over time in order to determine the reliability of metabolic rate prediction equations.

**Purpose:**

To evaluate the agreement, in terms of bias, absolute bias and accuracy between metabolic rate prediction equations and measured metabolic rate using indirect calorimetry system (IC), investigating also the factors affecting this agreement.

**Methods:**

The anthropometric features of 383 Caucasian participants of all Body Mass Index (BMI) classes were recorded and Resting Metabolic Rate (RMR) was measured by using the IC Fitmate portable device. The resulting values were compared with the predictive values of Harris & Benedict, Schofield, Owen, FAO-WHO-UNU, Mifflin and Harrington equations.

**Results:**

A closer approximation in agreement was obtained using the Harrington equation (based on BMI, age and gender). The equations using variables, such as weight, height, age and gender demonstrated higher agreement than the equations using merely weight and gender. Higher educational level was associated with normal weight, while higher calorific ratio was found in the class of normal-weighted individuals. An inverse relationship between ΒΜΙ and RMR was also observed and a logarithmic equation for calculating RMR was created, which was differentiated in relation to BMI classes, using the weight and gender variables.

**Conclusion:**

A better measurement agreement between RMR prediction equations and IC may be achieved due to BMI consideration. The present findings contributed to a better understanding of the measured parameters, confirming the inverse relationship between BMI and RMR. Age group and gender variables may also exert significant role on the bias response of some RMR equations.

**Electronic supplementary material:**

The online version of this article (10.1186/s12986-018-0278-7) contains supplementary material, which is available to authorized users.

## Background

Metabolic rate is the rate of energy expenditure in humans. The highest amount of this energy (50–75%) is essential for the development and maintenance of basic organic functions, while the person is at rest. Terms such as Basal Metabolic Rate (BMR) [1–4] or Resting Metabolic Rate (RMR) [5, 2] are used to define this energy expenditure and are often confused with each other, although they vary by approximately 10%. Basal Energy Expenditure (BEE) [6, 7] and Resting Energy Expenditure (REE) [8, 9] were derived from the conversion of BMR and RMR to kcal or kj /24 h. Several equations have been developed to calculate BMR and RMR, taking into account basic individual characteristics such as weight, height, age, gender etc., while they are measured using various methods of direct or indirect calorimetry (IC), or even using non-calorimetric methods. The rest of metabolic rate is the amount of energy expended for the individual’s physical activity and is expressed as Total Energy Expenditure (ΤΕΕ). TEE is the sum of BMR or RMR, taking also into account physical activity or exercise (20–40%), Thermic Effect of Food (TEF) which ranges between 5 and 30% and sometimes adaptive thermogenesis or/and Stress [9, 10].

In the present study, RMR was measured by using the IC Fitmate portable device, which is a functional solution in clinical and non-clinical environment. The reliability and quality aspect of this device has been investigated by a series of studies [11–13] and has been used in research to determine energy intake for various populations [14, 15]. However, the fact that these measuring devices are not always available necessitates the use of appropriate prediction equations, which are considered a useful tool for the calculation of metabolic rate. Several studies have investigated the reliability of prediction equations [16–18], as well as the factors that likely affect the body’s metabolic rate [19, 20] and thereafter the use of those factors for more accurate determination.

In view of the above considerations, the purpose of the present study was to evaluate the potential agreement in accuracy and absolute bias between prediction equations and measured values via the use of an IC device, to determine potential factors that affect the predictability of the equations and to create a new equation that may better respond to the study population.

## Study population and methods

### Study population

Data (Table 1) from 383 Caucasians participants were used in this study (men *n* = 105 and women *n* = 278), age 10–77 years old (mean: 37.5 ± 14.4 years old), Body Mass Index (BMI) 16.6–60.2 Kg/m^2^ (mean: 30.5 Kg/m^2^ ± 7.5 Kg/m^2^), weight range: 42.7–177 Kg (mean: 85.3 Kg ± 23.2 Kg) and height range from 1.44 up to 1.98 m (mean: 1.67 m ± 0.09 m). A few groups of participants were missing from the whole sample (Additional file 1: Table S1 AF): women > 60 years old in the normal weight class and men of age groups 10–18 and > 60 years old in the obese class II. Moreover, the under-weight class was not included in the statistical analysis due to the particularly low number of under-weight people in the sample (*n* = 3).

**Table 1:** Descriptive statistics of measured metabolic rates according to anthropometric factors of the subjects (*n* = 383)

Mean ± St.Dev (Lowest- Highest value)		
	Male	Female
Age	37.5 ± 15 (10–77)	37.5 ± 14 (12–76)
BMI^a^	32.0 ± 6.9 (16.6–57.8)	29.8 ± 7.6 (17.3–60.2)
Weight	100.1 ± 23.1 (59–177)	79.7 ± 20 (42.7–166)
Height	1.76 ± 0.08 (1.44–1.98)	1.63 ± 0.06 (1.48–1.86)

^a^RMRm means and their 95% confidence intervals in the BMI classes (kcal/kg body weight/day)

### REv1 sampling size

The sampling procedure followed the recording of first-time incomers that randomly or by date visited the laboratory so creating a physical succession of sampling numbers (identifications). A prospective study with the first 150 incomers showed reasonable results for the percentage deviation of RMR measured (RMRm) from RMR estimated (RMRe) but not for the statistics purposed for the breakdowns of physical variables. Therefore, we decided to over-double the sampling size for safety reasons.

## Methods

The anthropometric features were recorded, RMRm was read and RMRe was calculated by using Harris & Benedict (H-B) [21], H-B Rev. by Roza & Shizgal [22], H-B Abbreviated [23], Schofield [24], Owen [25], FAO-WHO-UNU (F-W-U) [26], Mifflin [27] and Harrington [28] prediction equations (Additional file 2: Table S1 AF) Prediction equations were grouped into 5 categories depending on the parameters they included (Additional file 2: Table S1 AF):

**Table 2:** Descriptive statistics and further relationships between RMRm (with IC) and RMRe

	Equation	Min	Max	Mean	St.dev	Mean bias	95% CI of bias	RMRm%	N
	RMRm	717	3189	1591	457.8				
Weight (wt), Height (ht), Age and Gender	H-B	1227	3227	1687	340.0	+ 96	62.2–129.3	6.0%	149
	H-B(Rev)	1213	3183	1678	338	+ 83	54.0–121.6	5.2%	143
	Mifflin	1242	2769	1718	260	+ 127	91.8–162.8	8.0%	129
Wt, Ht, Age Groups and Gender	F-W-U (1)	1124	3387	1849	571	+ 258	211.0–305.3	16.2%	125
Wt, Gender and Age Groups	F-W-U (2)	1124	3362	1695	336	+ 105	70.9–138.2	6.6%	145
	Schofield	1119	3364	1688	344	+ 97	62.0–132.3	6.1%	139
Wt, Age and Gender	Owen	1102	2684	1509	291	−82	−115.8- - 47.6	5.1%	131
	H-B (Abbr)	973	4248	1979	560	+ 388	343.5–432.8	24.4%	93
BMI, Age and Gender	Harrington	1166	2775	1627	283	+ 37	2.0–71.2	2.3%	153

*Mean bias*  RMRe-RMRm (individual estimates), *RMRm%* (absolute bias)*100/meanRMRm, *N* number of times RMRm% is ≤ 10%

Category 1- weight, height, age and gender (HB, HB Rev., Mifflin),

Category 2- weight, height, age group and gender (FWU),

Category 3- weight, gender and age group (FWU (2) and Schofield),

Category 4- weight and gender (Owen and HB Abbreviated).

Category 5- BMI, age and gender (Harrington).

All measurements were performed by a trained and certified nutritionist-dietitian under standard protocol. Body weight was measured and recorded by weight grading of 0.1 kg using Tanita fat monitor wearing no shoes and clothes. Height was measured by using the Seca height meter under standard protocol (no shoes, straight torso, having the buttocks, shoulders and head touching the vertical surface of the wall and looking horizontally). BMI was calculated in kg divided by the square of the height in m and the grouping was performed according to World Health Organizations guidelines. RMR was measured by using the indirect calorimeter Fitmate Pro (Cosmed), with silicone face mask, certified by Gold Standards. Scientific evidence indicates that in a steady state RQ is always in the range of 0.84 ± 0.04. Fitmate measures oxygen consumption Oxygen uptake (VO2), and gets a fixed breathing rate (RQ), which is set to 0.85 by default or can be set by the user. The measurement was performed after an overnight fast, absence of disease or infection and the least emotional disturbances within a quite environment (absence of noise) concerning people with normal nutritional status. Participants were requested not to engage in physical activity for 24 h, smoke, have coffee or stimulants for 12 h before the examination. Participants were also requested to lie on a mattress in the supine position for 20 min. The measurement was carried out for 12 min in a thermally neutral environment (22 °C up to 25 °C). The first 5 min of the measurement were skipped and were used for the last 7 min [9].

### Statistical analysis

RMRm data were compared with the corresponding values calculated from RMRe, according to the formula |mean(RMRm) – mean(RMRe)|, hence called absolute bias [29]. Percentage deviation of the estimated values (known as accuracy) was expressed as the absolute bias divided by mean(RMRe) and multiplied by 100. Percentage deviation of RMRm values was calculated in a similar manner (absolute bias divided by mean (RMRm) times 100). Higher values indicate lower accuracy and generally deviations less than 10% are indicative of adequate accuracy. Absolute biases of RMR (individual values) were examined against BMI classes, age groups and gender, using multiple regression analysis and backward elimination of variables importance. All absolute biases were transformed aiming to follow the normal distribution using λ transformed coefficients, which ranged between 0.34 and 0.41 (zone of logx and $\sqrt{\mathrm{X}}\Big)$. The physical characteristics expressed by age groups, gender and education level where cross-tabulated with BMI classes, using the Pearson’s chi square test of variable independence. Statistically significant differences were checked employing the standardized residuals (st. res). Values greater than |2| indicate significant effect. A regression equation was attempted between the dependent RMR of IC and the independent BMI-classes as literature indicates such relationship to hold [20, 30, 31]. All statistical analyses were performed by Minitab 17 statistical software (Minitab Inc., Pennsylvania, USA).

## Results

The comparisons between RMRm records with those of prediction equations are shown in Table 2. In all cases, the 95% confidence intervals of mean biases do not include zero, meant that there is not statistically significant agreement between the IC RMRm and the calculated RMRe. It should be kept in mind, however, that a close approximation towards a reasonable agreement is under consolidation after consulting the lower confidence intervals of biases of nearly all the equations concerned. This trend can be further improved by increasing the sample size and taking in parallel into account the effects of gender and age groups on the formation of the equations. The percentage deviation of either RMRm or RMRe was lower than the widely accepted 10% in 7 out or 9 equations Table 2. Harrington’s equation was found the most reliable according to the records of this study (2.3 and 2.2% correspondingly) while eqs. F-W-U (1) and H-B (Abbr) were the least acceptable.

The lowest bias and in close agreement was obtained using Harrington’s formula, based on BMI, age and gender (+ 37 units of deviation, CI range = 2.0–71.2) and the highest by H-B Abbreviation’s formula based on weight and gender (+ 388 Kcal). All deviations were overestimated (positive values) except for Owen’s formula (− 82 units, 5.1% accuracy) and ranged between − 82 and + 388 Kcal/day.

**Fig. 1:**
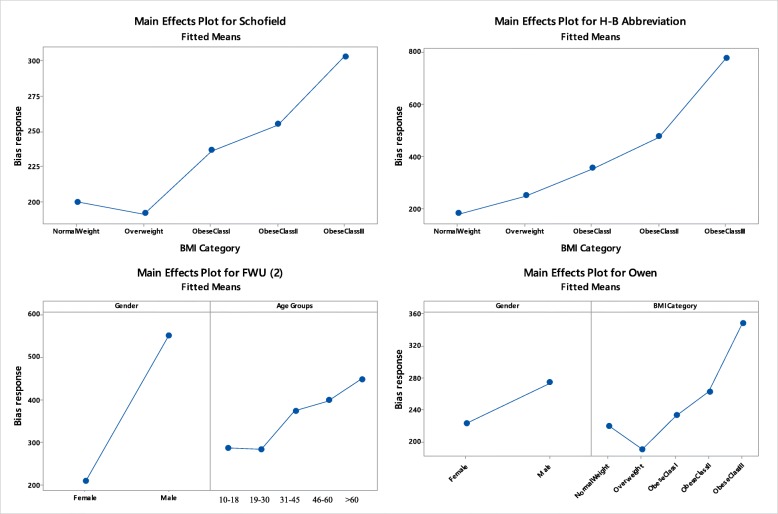
Main effects plots between transformed absolute bias response and the categorical variables, BMI-classes, Age-groups, Gender

Multiple regression analysis demonstrated that absolute bias responses are affected by only 4 prediction equations (Table 3): BMI by H-B Abbreviation, Owen and loosely by Schofield, Gender by F-W-U (2) and fairly by Owen, age group by F-W-U (2) equation. The effects are best elucidated in Fig. 1 in which, primarily, BMI increases the absolute bias with class increase, in particular, from obesity I and upwards. This increasing trend of bias response is smoothly depicted in the H-B Abbreviation equation along with all BMI classes, approaching a high magnitude of difference of 600 units (800–200). Males respond more vigorously in bias using F-W-U (2) equation than Owen’s. In the latter, the bias response steadily increases with age group after the point individuals reaching the age of 30–45 years old.

**Table 3:** Regression effects of gender, BMI and age on the absolute bias response

λ	Bias response	Gender	BMI	Age-group
0.41	H-B Αbbreviation		< 0.001	
0.41	Owen	0.036	< 0.001	
0.38	Schofield		0.023	
0.34	F-W-U (2)	< 0.001		0.023

The coefficient λ refers to optimal data transformation which approximates the square root

Statistically significant effects (dependencies) were found between BMI classes and gender, age groups and education level (Additional file 3: Table S3 AF). Males with normal weight are encountered less frequently than should be (st. res = − 2.74) and more frequently those with severe obesity (st. res = + 2.12). As for age groups, normal weight is rare for elders (> 60, st. res = − 2.14) and, interestingly, young people aged 10–18 appear more frequently severely obese (st. res = + 2.84) as also elders do so (st. res = + 2.12) while the group 19–30 years old occurs less frequently in the same class. Highly educated people (tertiary class) are encountered in higher numbers than expected when they cross-tabulate with normal weight (st. res = + 2.03).

**Fig. 2:**
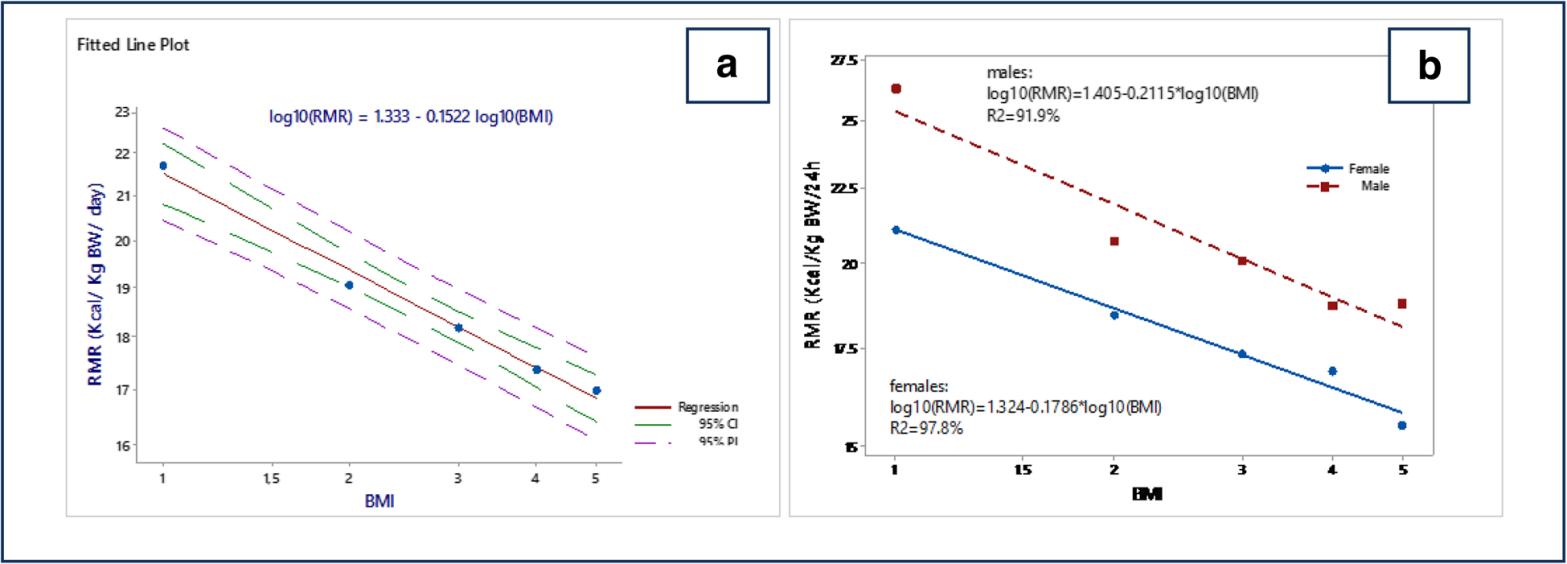
Log-linear relationship between RMRm and BMI-classes for the whole population (**a**) and according to gender (**b**)

BMI classes and estimated RMRm were further examined for linear trend using regression analysis after logarithmic transformation in order to conform to normal prerequisites (Fig. 2a). RMRm response declines linearly with body weight increase, starting from 21.7 Kcal/KgBW/day and ending down to 17.0 Kcal/KgBW/day (Table 4). Explicitly, more calories per Kg/BW are consumed by normal-weighted individuals than those belonging to higher BMI classes. Moreover, the decline is statistically significant between normal-weighted and over-weighted classes as indicated by the non-overlapping confidence limits (Table 4). The optimal regression line is described by the logarithmic (power) equation:

$$ \mathrm{RMR}\;\left(\mathrm{Kcal}/\mathrm{Kg}\;\mathrm{BW}/24\ \mathrm{h}\right)=21.53\;\mathrm{x}\;{\left(\mathrm{BMI}\right)}^{\hbox{-} 0.152}\left({\mathrm{R}}^2=98.9\%\right) $$

**Table 4:** RMRm means and their 95% confidence intervals in the BMI classes (kcal/kg body weight/day)

BMI classes	Mean	95% C.I	N
Normal Weight	21.7	20.8–22.6	84
Overweight	19.1	18.3–19.8	123
Obese Class I	18.2	17.3–19.1	77
Obese Class II	17.4	16.3–18.7	37
Obese Class III	17.0	15.9–18.1	59

or in linear form:

$$ {\log}_{10}\left(\mathrm{RMR}\right)=1.333-0.1522\;\mathrm{x}\;{\log}_{10}\left(\mathrm{BMI}\right) $$

The above equation explains that an increase of BMI by 1 class causes a decrease of RMR equal to 0.1522 Kcal/KgBW/day.

The above equation can be further expanded by separating the gender into males (Fig. 2b):

$$ {\displaystyle \begin{array}{l}{\mathbf{R}\mathbf{MR}}_{\mathrm{males}}\left(\mathbf{Kcal}/\mathbf{Kg}\;\mathbf{BW}/\mathbf{24}\ \mathbf{h}\right)=25.41\;\mathrm{x}\;{\left(\mathrm{BMI}\right)}^{\hbox{-} 0.2115}\left({\mathbf{R}}^{\mathbf{2}}=91.9\%\right)\ \mathrm{and}\\ {}{\log}_{10}\left({\mathrm{RMR}}_{\mathrm{males}}\right)=1.405-0.2115\;\mathrm{x}\;{\log}_{10}\left(\mathrm{BMI}\right)\end{array}} $$

and females (Fig. 2b):

$$ {\displaystyle \begin{array}{l}{\mathbf{R}\mathbf{MR}}_{\mathrm{females}}\left(\mathbf{Kcal}/\mathbf{Kg}\;\mathbf{BW}/\mathbf{24}\;\mathbf{h}\right)=21.09\;\mathrm{x}\;{\left(\mathrm{BMI}\right)}^{\hbox{-} 0.1786}\left({\mathbf{R}}^{\mathbf{2}}=97.8\%\right)\mathrm{and}\\ {}{\log}_{10}\left({\mathrm{RMR}}_{\mathrm{females}}\right)=1.324-0.1786\ \mathrm{x}\ {\log}_{10}\left(\mathrm{BMI}\right)\end{array}} $$

The configuration/formation of the above equations is depicted in Table 5.

**Table 5:** Stages of evolution of the proposed RMR equation

New equations in the initial form				
**log**_10_**(RMR)** = 1.333–0.1522 log_10_(ΒΜΙ) or **RMR (Kcal /24 h)** = 21.53 X (BMI)^-0.152^				
RMR equation for both sexes	**R**^2^ = 98.9%, n = 383 (males = 106, females = 277), age = 10–77 y.	BMI	Individual multiplications	
		Normal Weight = 1	(1)^-0.152^ = 0.848	(21,53 Χ 0,848) =18.26
		Overweight = 2	(2)^-0.152^ = 1.848	(21.53 X 1.848) = 39.79
		Obesity class I = 3	(3)^-0.152^ = 2.848	(21.53 X 2.848) = 62.32
		Obesity class II = 4	(4)^-0.152^ = 3.848	(21.53 X 3.848) = 82.85
		Obesity class III = 5	(5)^-0.152^ = 4.848	(21.53 X 4.848) = 104.38
**log10 (RMR)** = 1.324–0.1786 x log10(ΒΜΙ) or **RMR (Kcal/Kg BW/24 h)** = 25.41 x (BMI)^-0.2115^				
RMR equation for Males	**R**^2^ = 97.8%, n = 105 males, Age = 10–77 y	BMI	Individual multiplications	
	Normal Weight = 1	(1)^- 0.2115^ = 0.7885	(25.41 × 0.7885) =20.3	
	Overweight = 2	(2)^- 0.2115^ = 1.7885	(25.41 × 1.7885) = 45.50	
	Obesity class *I* = 3	(3)^- 0.2115^ = 2.7885	(25.41 × 2.7885) = 70.85	
	Obesity class II = 4	(4)^- 0.2115^ = 3.7885	(25.41 × 3.7885) = 96.26	
	Obesity class III = 5	(5)^- 0.2115^ = 4.7885	(25.41 × 4.7885) = 121.67	
**log**_10_**(RMR**) = 1.405–0.2115 x log_10_(ΒΜΙ) or **RMR (Kcal/Kg BW/24 h)** = 21.09 x (BMI)^-0.1786^				
RMR equation for Females	**R**^2^ = 91.9%, n = 278 females, Age = 10–77 y	BMI	Individual multiplications	
	Normal Weight = 1	(1) ^-0.1786^ = 0.8214	(21.09 × 0.8214) =17.32	
	Overweight = 2	(2) ^-0.1786^ = 1.8214	(21.09 × 1.8214) = 38.41	
	Obesity class I = 3	(3) ^-0.1786^ = 2.8214	(21.09 × 2.8214) = 59.50	
	Obesity class II = 4	(4) ^-0.1786^ = 3.8214	(21.09 × 3.8214) = 80.59	
	Obesity class III = 5	(5) ^-0.1786^ = 4.8214	(21.09 × 4.8214) = 101.68	

## Discussion

The design of this study and the determination of agreement between RMRm and RMRe was partly based on the methodology proposed by Michels, 2010 [29]. The present study compared the prediction equations based on particular factors that they use (weight, height, age, gender, BMI) and examined the predictive reliability of their responses. More specifically, the study supports findings of previous works that have shown that the use of BMI, which includes all the tissues of the body (via the use of weight divided by the square of height), renders the equations more reliable [20, 31]. The current study also supports previous findings, according to which characteristics such as gender, age, height and body weight are correlated with metabolic rate. These characteristics can easily be recorded and their significant effect on the reliability of equations has been indicated [20, 32].

In the present study, a close approximation but not significant was found concerning the agreement of the equations using weight, height and gender as variables, while a limitation by incorporating age groups as an additional factor of co-formulation was recorded. It has been supported in previous studies, that age has been considered as an important factor that increases the predictability of equations [33], further due to modifications of the individual anthropometric features that occur over time such as body composition [32, 34] and the organ and tissue metabolic rate [35, 36]. In addition, it has been suggested by other studies that the relationship between Free Fat Mass (FFM) and metabolic rate of older people could reflect the level of health of organism, as higher metabolic rates are associated with comorbidity and mortality [34, 37, 38]. Thus, factors correlated with organ and tissue metabolism, which vary depending on development, demonstrate a more significant effect on the metabolic capacity of organism [39]. Research also has supported the negative correlation between age and metabolic rate [31].

The results of our study were in good agreement with the findings of research that support the inverse relationship between educational level and BMI, such as NHANES 1999/2000 [40], WHO MONICA project [41], EPIC-PANACEA [42] and many others [43, 44]. The present study also showed consistently and significantly higher RMR values in people with lower BMI, especially in the normal weight class and these values are decreasing in a log-linear fashion (without taking into consideration the individual body composition analysis), enhancing the results of previous studies, which indicated that people with increased BMI exhibited lower RMR [45]. The proposed logarithmic equation was based on the BMI effect on the RMR responses taken from the IC device. Gender was also incorporated in the equation providing for both sexes reliable estimates (determined coefficients greater than 90%). Many studies have supported the fact that gender affects metabolic rate [20], due to the different allocation of FM [32] and FFM [32, 46], which also plays an important role in metabolic rate [47, 48]. It is also supported that hormonal factors lead to additional differences between genders [49]. However, RMR (BMI classes, Wt, and Gender based formulas) equations which have been created are not particularly interesting in terms of practical level of individual calculation, as their reliability is limited to a group level (BMI classes) and are initially used in order to develop a more reliable equation.

The data analysis of this study is going to be rescheduled in order to use a different statistic methodology based on the Bland-Altman graphical technique for the identification of the systematic difference between RMRm and RMRe. There will also be an effort to create a new equation which will approach RMRm at an individual calculation level.

### Limitations

Fitmate portable calorimetry device is used in the present study and has been proven through numerous studies that it generally provides a robust RMR assessment [50]. However, to avoid measurement errors, compliance with the protocols should be ensured [1]. Best practices prior to measurement include: the good health of the supervised person and the appropriate period of fasting, nicotine and caffeine abstinence and restriction of physical activity. During the measurement, best practices concern: the assurance of the appropriate environmental conditions of the test area (temperature, lack of noise), appropriate body position, rest period before the start of the measurement, time of day, but also the day is selected to be examined. From the use of the device, the causes that may alter the measurement are mainly: the duration of the measurement, the rejection time, the breathing rate (RQ) (which is set to 0.85 by default or can be set by the user and the type of gas collection devices (facemask, mouthpiece, canopy) [1].

It should be clarified that the use of silicone facemask used in the study lags behind the use of canopy. Moreover, possible oxygen losses, which may occur during the measurement, should be mentioned, if the auditee has a beard.

In addition, the RMR measurement system used for study purposes lags behind other more improved systems that allow a more accurate measurement of REE and respiratory quotient (RQ) by measuring VO2 (and carbon dioxide) and carbon dioxide production (VCO2) along with other respiratory parameters, as well as by calculation of the metabolic.

Furthermore, the RMR measurement period (time of year) was not recorded since variations in metabolic rate due to seasonal variations have not been reported in many studies, regarding people who live in a modern western environment and are not exposed to a cold climate. Finally, the absence of the thermogenic effect of brown adipose tissue during seasonal variations has yet not been reported and needs further investigation [51].

## Conclusion

The present study provides valuable information on the necessity of BMI consideration in the prediction equations of RMR, so allowing a better performance and a closer measuring agreement with various devices of metabolic interest. Additionally, our findings promote a better understanding of the measured parameters by increasing the predictive reliability of equations. The results confirm previous reports for the well-established inverse relationship between BMI and RMR and also the relatively higher calorific burden in the lower BMI classes which are strongly affiliated with the tertiary education level. Apart from BMI classes, variables such as Age Group and Gender play significant role on the absolute bias response of some RMR equations and need further investigation of their effects.

## Additional files

Additional file 1: **Table S1 AF.** Physical characteristics of the subjects as cross-tabulated by BMI classes, Age group and Gender. (DOCX 16 kb)

Additional file 2: **Table S2 AF.** Equations for Estimating Energy Expenditure. (DOCX 21 kb)

Additional file 3: **Table S3 AF.** Tabulated statistics between BMI classes and gender, age groups and education level. (DOCX 19 kb)

## Abbreviations

BMI: Body mass index
BMR: Basal metabolic rate
BMRe: Estimated BMR
BMRm: BMR measured
BW: Body weight
EE: Energy expenditure
FFM: Fat-free mass
FM: Fat mass
F-W-U: FAO-WHO-UNU
H-B: Harris-Benedict
IC: Indirect calorimetry
REE: Resting energy expenditure
RMR: Resting metabolic rate
RMRe: Estimated RMR
RMRm: Measured REE
TEE: Total energy expenditure
TEF: Thermic effect of food
