# Supplementary material for: Estimating the agreement between the metabolic rate calculated from prediction equations and from a portable indirect calorimetry device: an effort to develop a new equation for predicting resting metabolic rate
Source: Nutr Metab (Lond). 2018 Jun 15;15:41. doi: 10.1186/s12986-018-0278-7 (PMC6003108; doi:10.1186/s12986-018-0278-7)
Supplement: Supplementary file 1 — Table S1 AF. Physical characteristics of the subjects as cross-tabulated by BMI classes, Age group and Gender. (DOCX 16 kb) [file 12986_2018_278_MOESM1_ESM.docx]

| **Table 1 AF**  Physical characteristics of the subjects as cross-tabulated by BMI classes, Age group and Gender | | | | | | | | | | | | |
| --- | --- | --- | --- | --- | --- | --- | --- | --- | --- | --- | --- | --- |
| **Age Group** | | **10-18** | | **19-30** | | **31-45** | | **46-60** | | **>60** | |  |
| **Gender** | | **Female** | **Male** | **Female** | **Male** | **Female** | **Male** | **Female** | **Male** | **Female** | **Male** | **All** |
| **BMI classes** | **Underweight** | 1 | 0 | 2 | 0 | 0 | 0 | 0 | 0 | 0 | 0 | 3 |
|  | **Normal Weight** | 3 | 2 | 24 | 4 | 37 | 1 | 10 | 2 | 0 | 1 | 84 |
|  | **Overweight** | 4 | 3 | 33 | 8 | 31 | 14 | 16 | 8 | 5 | 1 | 123 |
|  | **Obese Class I** | 2 | 1 | 12 | 3 | 17 | 8 | 17 | 8 | 5 | 4 | 77 |
|  | **Obese Class II** | 2 | 0 | 4 | 3 | 9 | 4 | 8 | 3 | 4 | 0 | 37 |
|  | **Obese Class III** | 2 | 8 | 3 | 4 | 12 | 3 | 7 | 11 | 8 | 1 | 59 |
|  | **Total** | 14 | 14 | 78 | 22 | 106 | 30 | 58 | 32 | 22 | 7 | 383 |
