# Supplementary material for: Estimating the agreement between the metabolic rate calculated from prediction equations and from a portable indirect calorimetry device: an effort to develop a new equation for predicting resting metabolic rate
Source: Nutr Metab (Lond). 2018 Jun 15;15:41. doi: 10.1186/s12986-018-0278-7 (PMC6003108; doi:10.1186/s12986-018-0278-7)
Supplement: Supplementary file 2 — Table S2 AF. Equations for Estimating Energy Expenditure. (DOCX 21 kb) [file 12986_2018_278_MOESM2_ESM.docx]

| **Table 2 AF** Equations for Estimating Energy Expenditure | | | | | | | | | |
| --- | --- | --- | --- | --- | --- | --- | --- | --- | --- |
| **Parametrs** | **Predictive equations** | **Population Description** | | **Αge** | **R^2^**  **^Male^** | **R^2^**  **^Female^** | **Male** | | **Female** |
| **Weight (Wt), Height (Ht), Age and Gender** | **Harris–Benedict in kcal/d (1919)** | n=239,white normal-weight, 16-63y,  136 males (M) (weight mean 61.1 ±10.3 Kg and mean ages27±9 y.)  103 females (F) (mean weight 56.5±11.5 Kg and mean ages31±4y.),  Over a ten y. period | | adult | 0.75 | 0.53 | (13.75 x wt ) + (5,003 x ht ) − (6.755 x age ) + 66.47 | (9.563 × wt) + (1.850 × ht) – (4.676 × age) +  655.1 | |
|  | **H-B Rev. by Rosa & Shizgal  in kcal/d (1984)** | n = 337, 168 M.  169 F., (with wider age range from original H-B) | | adult | 0.88 | 0.77 | (13.397 x wt) + ( 4.799 x ht)-(5.677 x age) + 88.362 | (9.247 x wt) + ( 3.098 x ht)-(4330 x age) + 447.593 | |
|  | **Mifflin in kcal/d (1990)** | n = 498,  19-78 y (mean ages 44±14 y)  251 M (mean weight 87.5 ±14.4 Kg )  247 F (mean weight 70.2±14.1 Kg | | adult | 0.71 | | (9.99 x wt) + (6.25 x ht) – (4.92 x age) + 5 | (9.99 x wt) + (6.25 x ht) – (4.92 x age) – 161 | |
|  |  | n = 11.000. Many Ethnic groups and broad BMI range | | 10-18 |  |  | (16.6 x wt) – (77 x ht) + 572 | (7.4 x wt) + (482 x ht) + 217 | |
| **Wt, Ht , Age Groups and Gender** | **FAO/WHO/UNU in kcal/d (1985)** |  |  | 18– 30 | 0.42 | 0.53 | (15.4 x wt) – (27 x ht) + 717 | (13.3 x wt) + (334 x ht) + 35 | |
|  |  |  |  | 30-60 | 0.36 | 0.49 | (11.3 x wt) + (16 x ht) + 901 | (8.7 x wt)– (25 x ht) + 865 | |
|  |  |  |  | >60 | 0.71 | 0.67 | (8.8 X wt) + (1128 X ht) -1072 | (9,2 X wt0+ (637 X ht) -302 | |
| **Wt, Gender and Age Groups** | **FAO/WHO/UNU in kcal/d (1985)** | n = 11.000. Many Ethnic groups and broad BMI range | | < 3 | 0.97 | 0.97 | (60.9 × wt ) - 54 | (61. 3 × wt ) - 51 | |
|  |  |  |  | 3–10 | 0.86 | 0.85 | (22.7 × wt ) + 495 | (22.43 × wt ) + 499 | |
|  |  |  |  | 10–18 | 0.90 | 0.75 | (17.5 × wt ) + 651 | (12.2 × wt ) + 746 | |
|  |  |  |  | 18–30 | 0.65 | 0.72 | ( 15.3× wt ) + 679 | (14.7× wt ) + 496 | |
|  |  |  |  | 30–60 | 0.60 | 0.70 | ( 11.6 × wt ) + 879 | (8.7 × wt ) + 829 | |
|  |  |  |  | > 60 | 0.79 | 0.74 | ( 13.5× wt ) + 487 | ( 10.5× wt ) +596 | |
|  |  | n | n |  |  |  |  |  | |
|  | **Schofield**  **in kcal/d (1985)**  n=7173, of whom the 3396 Italians  3-60 y, 4809 M,  2364 F | 162 | 137 | < 3 |  |  | (59.512 × wt ) - 30.4 | (58.317 × wt) - 31.1 | |
|  |  | 338 | 413 | 3–10 |  |  | (22.706 × wt ) + 504.3 | (20.315 × wt) + 485.9 | |
|  |  | 734 | 575 | 10–18 |  |  | (13.384 × wt) + 692.6 | (17.686 x wt ) +658.2 | |
|  |  | 2879 | 829 | 18–30 | 0.42 | 0.53 | (15.057 x wt) + 692.2 | (14.818 × wt) + 486.6 | |
|  |  | 646 | 372 | 30–60 | 0.36 | 0.46 | (11.472 x wt) +873.1 | (8.126 × wt) + 845.6 | |
|  |  | 50 | 38 | > 60 | 0.50 | 0.46 | (11.711 x wt) +587.7 | (9.082 × wt) + 658.5 | |
| **Wt and Gender** | **Owen in kcal/d**  **(1986 & 1987)** | n = 60 M, Caucasian, Negro, and Oriental  volunteers 18-82 y, 60-17 1 kg  n = 44 F (included 8 athletes), No specific racial/ethnic information provided, 18-65 y, 43-143 kg. | | adult | 0.71 | | 879 + 10.2 x wt | 795 + 7.18 x wt | |
|  | **H-B Abbreviated in kcal/d** | Version for persons with normal height and weight | | adult |  |  | (wt x 1kcal/kg) x 24 hr | (wt x 0.95 kcal/kg) x 24 hr | |
| **BMI , Age and Gender** | **Harrington in kcal/d (1997)** | Same population as Mifflin | | adult | 0.62 | | (BMI x 28,15) – (age x 6,44) + 1290 | (BMI x 28.15) – (age x 6.44) + 905 | |
| A = age (yrs); Sex= 1 for males and 0 for females; W = actual wt(kg); Height in m; Fat Free Mass ( FFM) and Fat MASS (FM) in kg; O=obesity( if present=1, absent=0) | | | | | | | | | |
