# Supplementary material for: Estimating the agreement between the metabolic rate calculated from prediction equations and from a portable indirect calorimetry device: an effort to develop a new equation for predicting resting metabolic rate
Source: Nutr Metab (Lond). 2018 Jun 15;15:41. doi: 10.1186/s12986-018-0278-7 (PMC6003108; doi:10.1186/s12986-018-0278-7)
Supplement: Supplementary file 3 — Table S3 AF. Tabulated statistics between BMI classes and gender, age groups and education level. (DOCX 19 kb) [file 12986_2018_278_MOESM3_ESM.docx]

| **Table 3 AF** Tabulated statistics between BMI classes and gender, age groups and education level | | | | | | | | | |  |
| --- | --- | --- | --- | --- | --- | --- | --- | --- | --- | --- |
| **BMI *v*s gender** | | | | | | | | | |  |
|  | | **Female** | **Male** | **All** | | | | | | |
| **1** | | 74 | 10 | 84 | | | | | | |
|  | | 1.6944 | **-2.7421** |  | | | | | | |
| **2** | | 89 | 34 | 123 | | | | | | |
|  | | -0.0014 | 0.0023 |  | | | | | | |
| **3** | | 53 | 24 | 77 | | | | | | |
|  | | -0.3649 | 0.5905 |  | | | | | | |
| **4** | | 27 | 10 | 37 | | | | | | |
|  | | 0.0432 | -0.0700 |  | | | | | | |
| **5** | | 32 | 27 | 59 | | | | | | |
|  | | -1.6371 | 2.6494 |  | | | | | | |
| **All** | | 275 | 105 | 380 | | | | | | |
| Pearson Chi-Square = 20.578; DF = 4; P-Value = 0.000 | | | | | | | | | |  |
| **-------------------------------------------------------------------------------------------------** | | | | | | | | | |  |
| **BMI *v*s age** | | | | | | | | | |  |
|  | | **10-18** | **19-30** | **31-45** | | **46-60** | | **>60** | **All** |  |
| **1** | | 5 | 28 | 38 | | 12 | | 1 | 84 |  |
|  | | -0.396 | 1.361 | 1.448 | | -1.770 | | **-2.137** |  |  |
| **2** | | 7 | 41 | 45 | | 24 | | 6 | 123 |  |
|  | | -0.588 | 1.647 | 0.148 | | -0.951 | | -1.105 |  |  |
| **3** | | 3 | 15 | 25 | | 25 | | 9 | 77 |  |
|  | | 1.056 | -1.090 | -0.487 | | 1.584 | | 1.289 |  |  |
| **4** | | 2 | 7 | 13 | | 11 | | 4 | 37 |  |
|  | | -0.388 | -0.823 | -0.067 | | 0.756 | | 0.700 |  |  |
| **5** | | 10 | 7 | 15 | | 18 | | 9 | 59 |  |
|  | | **2.837** | **-2.106** | -1.331 | | 1.077 | | **2.119** |  |  |
| **All** | | 27 | 98 | 136 | | 90 | | 29 | 380 |  |
| Pearson Chi-Square = 45.523; DF = 16; P-Value = 0.000 | | | | | | | | | |  |
| **--------------------------------------------------------------------------------------------------** | | | | | | | | | |  |
| **BMI vs education** | | | | | | | | | |  |
|  | **Primary** | | **Secondary** | | **Tertiary** | | **All** | | | |
| **1** | 7 | | 28 | | 49 | | 84 | | | |
|  | -0.3396 | | -1.8090 | | **2.0314** | |  | | | |
| **2** | 7 | | 60 | | 56 | | 123 | | | |
|  | -1.3630 | | 0.3141 | | 0.3095 | |  | | | |
| **3** | 9 | | 39 | | 29 | | 77 | | | |
|  | 0.6314 | | 0.4881 | | -0.7995 | |  | | | |
| **4** | 4 | | 18 | | 15 | | 37 | | | |
|  | 0.2642 | | 0.1606 | | -0.2893 | |  | | | |
| **5** | 9 | | 33 | | 17 | | 59 | | | |
|  | 1.4426 | | 1.0202 | | -1.7282 | |  | | | |
| **All** | 36 | | 178 | | 166 | | 380 | | | |
| Pearson Chi-Square = 17.130; DF = 8; P-Value = 0.029  Standardized residuals with values greater than \|2\| are shown in bold. | | | | | | | | | |  |
|  | | | | | | | | | |  |
